# Transcutaneous auricular vagus nerve stimulation as a potential therapy for attention deficit hyperactivity disorder: modulation of the noradrenergic pathway in the prefrontal lobe

**DOI:** 10.3389/fnins.2024.1494272

**Published:** 2024-12-04

**Authors:** Jincao Zhi, Shiwen Zhang, Meiling Huang, Huan Qin, He Xu, Qing Chang, Yan Wang

**Affiliations:** ^1^Graduate School of Heilongjiang University of Chinese Medicine, Harbin, China; ^2^Clinical Medical College of Acupuncture Moxibustion and Rehabilitation, Guangzhou University of Traditional Chinese Medicine, Guangzhou, China; ^3^Department of Rehabilitation Medicine, The First Affiliated Hospital of Xi’an Jiaotong University, Xi’an, China; ^4^Rehabilitation Center, The Second Affiliated Hospital of Heilongjiang University of Traditional Chinese Medicine, Harbin, China

**Keywords:** attention deficit hyperactivity disorder, transcutaneous auricular vagus nerve stimulation, prefrontal lobe, locus coeruleus, norepinephrine

## Abstract

Attention deficit hyperactivity disorder (ADHD) is a neurodevelopmental disorder characterized by developmental impairments, inattention, motor hyperactivity, and impulsivity. Currently, there is no effective intervention that can completely cure it. One of the pathogenic mechanisms of ADHD involves abnormalities in the norepinephrine (NE) pathway within the prefrontal cortex (PFC). In recent years, transcutaneous auricular vagus nerve stimulation (taVNS), a non-invasive neuromodulation technique, has demonstrated promising potential in the treatment of neurological and psychiatric disorders. However, its application in the management of ADHD remains relatively unexplored. Previous studies have shown that taVNS exerts therapeutic effects on attention, cognition, arousal, perception, and behavioral regulation primarily through activating the vagus nerve conduction pathway, specifically targeting the nucleus tractus solitarius - locus coeruleus - NE pathway. These findings have led to the hypothesis that taVNS may be an effective intervention for ADHD, with NE and its pathway playing a pivotal role in this context. Therefore, this review comprehensively examines the correlation between NE pathway alterations in the PFC and ADHD, the mechanism of action of taVNS, and the potential role of the NE pathway in treating ADHD with taVNS, aiming to provide a theoretical foundation for clinical applications.

## 1 Introduction

Attention deficit hyperactivity disorder (ADHD) is a neurodevelopmental disorder characterized by developmental impairments, inattention, motor hyperactivity, and impulsivity, with a male to female diagnostic ratio of approximately 4:1, occurring prevalently in childhood (Thapar and Cooper, 2016; Martin, 2024). According to a systematic evaluation and meta-analysis of studies on the global prevalence of ADHD among children and adolescents in the year 2023, the prevalence rate was 7.6% (95% confidence interval: 6.1–9.4%) among children between the ages of 3 and 12 years and 5.6% (95% confidence interval: 4.8–7%) among adolescents between the ages of 12 and 18 years (Salari et al., 2023). Existing research suggests that the symptoms and deficits of ADHD tend to reveal themselves before school age, and although the core symptoms of ADHD attenuate with age, they persist into adulthood in approximately two-thirds of cases (Van Meter et al., 2024). According to the 2021 World ADHD Federation International Consensus Statement, ADHD affects 5.9% of adolescents and 2.5% of adults worldwide (Li et al., 2024). Although the pathogenesis of ADHD has not yet been clarified, existing studies suggest that it is caused by a synergistic combination of various genetic-environmental factors (Faraone et al., 2021; Demontis et al., 2023; Kleppesto et al., 2024). Furthermore, these studies indicate that the pathogenesis and development of ADHD are closely related to several genetic, neurodevelopmental, familial, and social factors. The theory of abnormal central neurotransmitter function is the current important hypothesis for the pathogenesis of ADHD, which mainly includes the dopamine (DA)/norepinephrine (NE) hypoactive state. Meanwhile, ADHD is a highly heterogeneous disorder that frequently combines other psychiatric and somatic comorbidities leading to poor functional outcomes, including interpersonal aspects, high risk of unintentional injuries, significant problems with substance use, risk-taking behaviors, unintended pregnancies, and self-harm, which place a heavy burden on individuals, families, and society (Taipale et al., 2024).

Treatment of ADHD usually involves multimodal therapies, including a combination of psychoeducational, parent/teacher training, and pharmacological treatments that can alleviate the symptoms associated with ADHD, but there is currently no effective method to eradicate ADHD (Faraone et al., 2024; Van Vyve et al., 2024). Pharmacological, psychosocial, and behavioral interventions remain the primary evidence-based treatments in existing research (Mechler et al., 2022; Faraone et al., 2024). Furthermore, ADHD has long been conceptualized as a neurobiological disorder of the prefrontal cortex (PFC) and its connections (Durston et al., 2011). Although short-term pharmacological treatments (e.g., MPH, atomoxetine, and possibly guanfacine) can increase levels of DA and NE in the PFC, improving ADHD symptoms and co-morbidities. However, clinical studies have shown that such pharmacological interventions may also cause a variety of side effects, including somnolence, sleep disturbances, decreased appetite, and mood disorders (Brikell et al., 2024; Vertessen et al., 2024). Notably, transcutaneous auricular vagus nerve stimulation (taVNS), as an emerging non-invasive adjunctive therapy to modulate brain physiology by electrically stimulating the vagus nerve (VN) distribution area on the surface of the auricle, overcomes the limitations of the traditional vagus nerve stimulation (VNS) procedure, which has led to the innovation and development of VNS (Gerges et al., 2024; Zou et al., 2024). Numerous clinical studies have observed that taVNS can improve cognitive dysfunction in patients with epilepsy (Zhang et al., 2024), impaired consciousness (Wang L. et al., 2023), stroke (Du et al., 2024), and depression (Liu et al., 2024). The neurophysiological mechanisms underlying its occurrence are mainly mediated by the nucleus tractus solitarius (NST) - locus coeruleus (LC) - NE pathway as a key target for its functioning, and this pathway is important for attention, cognition, arousal, perception, and behavioral modulation (Groves and Brown, 2005). Based on these studies, a hypothesis has been formulated suggesting that taVNS may serve as an effective intervention for the management of ADHD, with NE and its pathways playing a pivotal role in this context.

## 2 Correlation between NE system in PFC and ADHD

### 2.1 Correlation between PFC and ADHD

The PFC is located in the anterior frontal lobe of the brain, anterior to the motor cortex. It is often referred to as the brain’s command and control center, which receives and integrates diverse information from all regions, both internal and external, through tightly connected neural circuits (Stevens, 2010). The PFC is involved in higher-order cognitive functions, and its neural circuits possess a unique ability to enable PFC neurons to co-activate and sustain information even when the stimulating stimuli are no longer present in the immediate environment. However, when confronted with interference, the PFC engages its unique “working memory” (WM) mechanism, activating PFC neurons to direct overt responses (movements), to covert responses (attention), and to suppress inappropriate reactions (Chafee and Heilbronner, 2022). In addition to this, the left anterior cingulate cortex/medial prefrontal cortex is responsible for inhibitory control, and the bilateral middle frontal gyrus/precentral gyrus is responsible for modulating dorsal and ventral attention networks, while playing a key role in the flexible regulation of endogenous and extrinsic attention (Wang et al., 2024). Thus, the honeycomb-like neuronal network specific to the PFC can use information from WM to manage stability and ensure content coherence in pursuit of attentional goals, execute plans, and organize appropriate actions (Arnsten, 2011; Yan and Rein, 2022).

**FIGURE 1:**
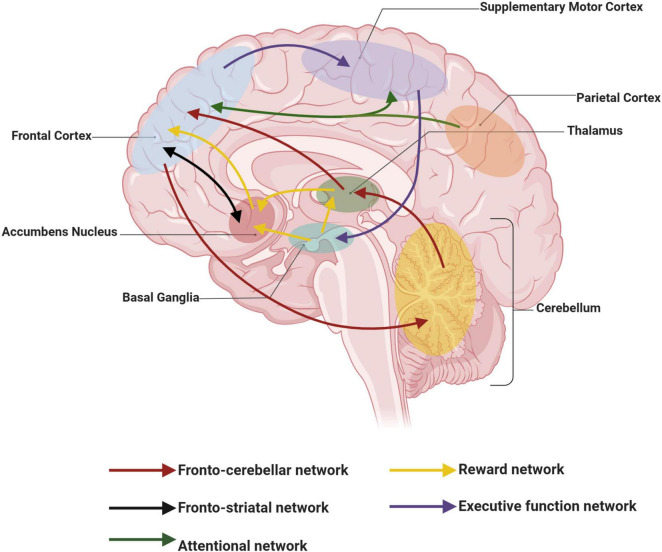
Schematic representation of functional networks involved in the pathophysiology of attention deficit hyperactivity disorder (ADHD). Curves of the same color connect functional networks that are identical. This figure was created with BioRender.com.

The core symptoms of ADHD stem from disrupted circuits of attention regulation and action inhibition. Functional abnormalities in the neural networks of the PFC in ADHD patients (reduced PFC gray matter, diminished PFC connectivity, and altered PFC function) all impair cognitive control of behavior and attention (Arnsten, 2010). The associations of functional circuits involved in the pathophysiology of ADHD are shown in Figure 1. Thus, the neuronal pathways associated with the PFC in ADHD appear to have a central role, primarily manifesting in the following aspects.

First, dysfunction of the prefrontal-subcortical pathway is recognized as one of the core pathophysiological mechanisms of ADHD (Halperin and Schulz, 2006). Structural and functional imaging studies have demonstrated significant abnormalities in neuronal connectivity, signaling, and metabolic activity in the PFC of ADHD patients (Cubillo et al., 2010; Del Campo et al., 2011). For example, a structural magnetic resonance imaging (MRI) study of ADHD adults demonstrated that these people have volume differences in brain regions involved in attention and executive control, primarily in the overall cortical gray matter, with significantly smaller PFC volumes (Seidman et al., 2006). Additionally, diffusion tensor imaging reveals a decreased anatomical functional connectivity of white matter tracts linking the PFC to other brain regions in ADHD patients (Curatolo et al., 2010). Meanwhile, resting-state fMRI analyses showed a more decentralized pattern of connectivity in the default mode and frontal control network in ADHD children, mainly in terms of increased functional connectivity in the right inferior frontal gyrus and bilateral medial frontal lobes, which may reflect a reduced or delayed functional segregation of the PFC (Bos et al., 2017). Therefore, the inability of the PFC to effectively regulate the functions of posterior cortical and subcortical structures in ADHD patients may underlie their abnormalities in attention, emotional regulation, and behavioral control.

Second, the developmental process of the PFC is also closely related to the onset of ADHD (Halperin and Schulz, 2006). Moreover, the development of the PFC involves neuron generation, migration, differentiation, and synapse formation. The PFC is the last to develop in the central nervous system (CNS) and matures at the latest in individual development. However, developmental impairment of PFC in ADHD patients, including structural and functional abnormalities, is evident early in life (Sullivan and Brake, 2003). In addition, functional near-infrared spectroscopy (fNIRS) has shown that children with ADHD exhibit lower PFC activation in performing functional tasks compared to their peers who have developed normally throughout childhood (Su et al., 2023). Shaw and colleagues (Shaw et al., 2007, 2012) utilized MRI to assess the developmental trajectory of brain morphology in ADHD patients, revealing delayed cortical thickness development in both pediatric and adult patients compared to controls. Notably, this delay was evident as cortical thinning, particularly in the frontal, parietal, temporo-parietal, and occipital regions, highlighting the slowed or impaired development of the right lateral PFC.

In addition, interactions between the PFC and other brain regions also play an important role in the pathogenesis of ADHD. Extensive connections between the PFC and neural circuits in the parietal cortex, basal ganglia, cerebellum, hippocampus, and corpus callosum have been identified through functional imaging studies, which together participate in ADHD-associated functional networks (Curatolo et al., 2009). However, in ADHD patients, the interactions between these brain regions may be abnormal. Significantly reduced connectivity in the bilateral lateral PFC, anterior cingulate cortex, superior parietal lobule, and cerebellum has been reported in adults with ADHD compared with healthy controls (Wolf et al., 2009). ADHD patients exhibit reduced interregional functional connectivity between the right inferior frontal lobe, frontal striatum, and frontal-parietal neural networks in a cessation task (Cubillo et al., 2010). In summary, there is a close correlation between the PFC and ADHD. Therefore, understanding the molecular and cellular regulation that modulates PFC function is crucial for exploring therapeutic approaches to ADHD and developing targeted medications, ultimately leading to better patient outcomes.

### 2.2 Effects of NE signaling pathways on PFC function

The PFC network is capable of guiding action in the absence of bottom-up external stimuli and through the mutual excitation of a network of interconnected pyramidal neurons on dendritic spines to store goals/rules (Arnsten, 2011; Vogel et al., 2022). However, the activity of PFC networks is largely dependent on a correct neurochemical milieu, where arousal pathways coordinate cognitive states with environmental events (Arnsten, 2011).

**FIGURE 2:**
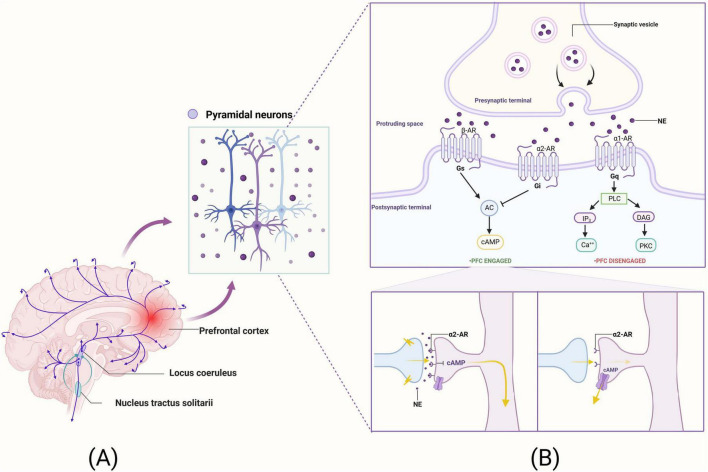
The neural pathways of NE within the brain and its mechanistic roles in synaptic functions of pyramidal neurons in the PFC. **(A)** The operating pathway of the NE system in the brain. **(B)** The mechanistic underpinnings of NE’s action within the synapses of pyramidal neurons in the PFC. This figure was created with BioRender.com.

The most representative arousal pathway, the NE pathway, has neurons that originate in the LC within the brainstem and project terminally to a wide range of brain regions, including the PFC. The operating pathway of the NE system in the brain is shown in Figure 2A. Anatomically, the LC provides the sole NE neural input to the PFC, and the PFC is one of the few cortical sites that provide cortical information to the LC. The NE release levels may rapidly alter the strength of PFC network connections to coordinate cognitive states with physiological demands (Yan and Rein, 2022). Among other things, NE is also released in the PFC in response to our arousal state, with an inverted U-shaped dose effect on PFC cognitive function (Levy, 2009). In this inverted U-shaped effect, either too little NE (such as depletion or fatigue) or too much NE (like uncontrollable stress) impairs PFC function, while intermediate levels of NE, released when subjects are alert or interested, reinforce and shape inputs to optimize PFC function (Arnsten, 2011). Thus, the level of NE released in the PFC can harmonize brain arousal states and PFC function.

However, the effects of NE on arousal, mood, and behavior are mediated through interactions with a wide range of receptors. Based on the pharmacological profile of adrenergic receptors, which include those responsive to NE, they can be classified into two major groups: α and β receptors. These are divided into three major subgroups each: α1 (α1A, α1B, α1D), α2 (α2A, α2B, α2C), and β (β1, β2, β3), due to differences in coupled G proteins, signaling, and effector systems (Perez, 2020).

Norepinephrine has the highest affinity for α2 receptors, followed by α1 receptors, and the lowest affinity for β receptors (Ramos and Arnsten, 2007). Among them, α2A receptors play an important role in PFC cognitive function. Furthermore, robust synaptic connections enable the PFC to more effectively enhance WM, engage in the regulation of attentional control, and modulate behavioral inhibition. The efficacy of PFC network connectivity relies on NE stimulation of α2A receptors located on the spines of PFC pyramidal cells (Arnsten, 2011). α2A receptors are located on dendritic spines in the vicinity of the ion channel and control the effects of synaptic inputs on the spines. When there is no α2A receptor stimulation, cyclic adenosine monophosphate (cAMP) levels are high, potassium channels (HCN channels) open, nearby synaptic inputs are diverted, and incoming information escapes, weakening synaptic connections (Arnsten and Jin, 2012). Conversely, when α2A receptors are stimulated by NE or guanfacine (α2A receptor agonists), activation of Gi leads to a decrease in cAMP levels, which in turn reduces the opening of HCN channels and enhances the efficacy of neural network inputs and synaptic excitatory inputs, thereby promoting neuronal firing activity and PFC function (Arnsten and Jin, 2012; Joyce et al., 2024). The mechanism of action of the NE system at the synapses of PFC pyramidal neurons is illustrated in Figure 2B.

## 3 Effects of ta-VNS on PFC-related adrenergic pathways

Invasive VNS is a surgical procedure in which electrodes are placed directly on the VN. Although its therapeutic effects have been demonstrated in clinical settings, invasive VNS has common side effects due to its invasive nature (including hoarseness, wound infections, shortness of breath, and coughing) (Brauer et al., 2023). Consequently, taVNS has been developed as a non-invasive, cost-effective, and easily applicable alternative, exerting its therapeutic effects through the application of low-frequency pulsed current to stimulate the peripheral branch of the VN in the auricle (Hilz, 2022).

### 3.1 Anatomical basis of the auricular branch of the vagus nerve

**FIGURE 3:**
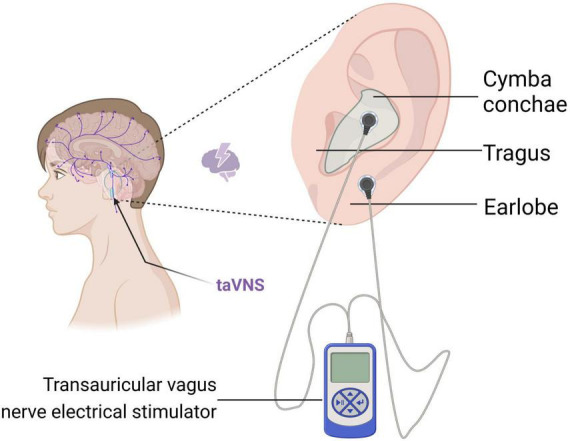
Schematic representation of the conduction of the NE system in the brain after taVNS stimulation. This figure was created with BioRender.com.

The VN is the 10th pair of cranial nerves, the longest and most widely distributed pair of cerebral nerves. This nerve is a mixed nerve composed of 20% efferent fibers and 80% afferent fibers, encompassing both sensory and motor nerve fibers (Butt et al., 2020). The sensory neurons in this nerve protrude centrally into the brainstem, terminating at the NST (nucleus of the solitary tract) and the nucleus of the spinal tract of the trigeminal nerve. Additionally, the motor neurons originate from the nucleus ambiguus of the medulla oblongata and the dorsal nucleus of the VN. Among them, the auricular branch of the VN is mainly distributed in the tragus, the cymba conche, and the cavum conche. It crosses the jugular foramen via the auricular branch of the VN, then enters the medulla oblongata, and subsequently ascends through the trigeminal nucleus of the spinal cord to connect with the NST (Ellrich, 2019; Kaniusas et al., 2019a). Yakunina et al. (2017) conducted a study using fMRI and showed that the cymba conche has the strongest activating effect on the vagal pathway and is considered to be the optimal therapeutic site for taVNS. The conduction diagram of the NE system in the brain after taVNS stimulation is shown in Figure 3. Furthermore, the left and right VN in humans provide parasympathetic visceromotor innervation to the sinoatrial node and the atrioventricular node, respectively (Kaniusas et al., 2019b). Consequently, to prevent intracardiac conduction abnormalities from inducing arrhythmias, clinical practice often favors the application of taVNS on the left ear (Chen et al., 2023b; Zhang et al., 2023; Zhou et al., 2023). Therefore, the activation of the central nervous system through stimulation of VN sensory fibers distributed in the periphery serves as the theoretical basis for the treatment of neurological disorders using taVNS.

### 3.2 taVNS activates the NST-LC-NE system

The NST is considered to be the relay station between the afferent fibers of the VN and the associated nuclei in the brain. It receives the majority of afferent fibers from the VN and a minor portion of information from other peripheral regions, subsequently integrating and projecting this sensory information to higher centers (Wang L. et al., 2023). The projection fibers of NST form three main pathways: (i) they form an autonomic feedback loop; (ii) they project directly to the medullary reticular formation; and (iii) they project to the pontine parabrachial nucleus, the LC, and other structures in the brain. Of these, the NST receives approximately 95% of the VN input and projects directly to the LC via either excitatory or inhibitory pathways. The LC plays a significant role as a mediator in the reticular superior activation system. It projects to the cerebral cortex through several key structures, including the nucleus of the median eminence, the amygdala, the hypothalamus, the orbitofrontal cortex, and the cingulate gyrus (Maness et al., 2022). Consequently, NE released from this region exerts an excitatory effect on the cerebral cortex. Furthermore, an elevated NE concentration within the LC activates the PFC and a broad network of cortical and subcortical circuitry projections throughout the brain (Ludwig et al., 2021). This activation leads to extensive remodeling of brain networks in areas densely innervated by the LC, facilitating enhanced task performance, such as vigilance and attention (Briand et al., 2020).

The fMRI scans showed that taVNS promoted sequential activation of the NST and LC, as well as brain regions closely related to cognitive functions with which these structures have fiber contact (Borgmann et al., 2021). Additionally, taVNS produced more activation in brainstem regions, including the LC. In an animal model, a 2-week VNS intervention significantly increased extracellular NE levels in the PFC and hippocampus and enhanced tonic activation of postsynaptic α2A in pyramidal neurons (Manta et al., 2013). And taVNS significantly increased c-Fos protein expression in the NST and LC nuclei (Yue et al., 2023). Taken together, given that both NST and LC are key nodes for the regulatory effects of taVNS, taVNS may be involved in the modulation of cognitive functions such as attention, arousal, behavior, and memory through this pathway.

Relevant research indicates that the activation of the NE system is determined by the combination of stimulus intensity and pulse width, rather than solely by intensity alone (Hulsey et al., 2017). In previous clinical studies, a stimulus intensity of 0.5 mA, frequencies of 25 Hz/20 Hz, and pulse widths ranging from 0.25 to 1 ms have been most commonly employed as stimulation parameters for taVNS (Sun et al., 2023; Yang et al., 2023; Pan et al., 2024). However, it remains uncertain whether the activation of the NST-LC-NE system via taVNS requires the adjustment of these parameters to achieve optimal stimulation effects. Notably, Sclocco et al. (2020) compared four different stimulation frequencies (2, 10, 25, and 100 Hz) and found that taVNS at 100 Hz elicited the highest fMRI response within the NST-LC system. However, further clinical research is needed to determine whether this frequency provides the greatest therapeutic benefit for ADHD and to assess tolerability among pediatric patients.

## 4 taVNS as potential evidence for the treatment of ADHD

Attention deficit hyperactivity disorder is characterized by core symptoms including impaired impulse control, increased motor activity, and disrupted attention regulation, which originate from disruptions in the circuits underlying attentional control and motor inhibition. Using behavioral and optogenetic techniques, Bari et al. (2020) found that LC neuron stimulation increased goal-directed attention and decreased impulsivity, while its inhibition exacerbated distraction and increased impulsive responses. Interestingly, they also found that attention and response inhibition were controlled by two different projections from the LC: one directed to the dorsomedial PFC and the other to the ventral lateral orbitofrontal cortex. So activation of the LC-NE system is thought to be involved in attention regulation and behavioral control by modulating neuronal activity in the PFC. However, the LC-NE system’s involvement is further supported by the established close connection between the VN afferent pathway and the structures in the brain that transmit NE; NE may act at different levels (synapses, cells, microcircuits, and networks) and modulate PFC activity. Thus, taVNS has been proposed as a method to facilitate attention and behavioral modulation in ADHD patients, with the LC-NE system being a potential key factor in this process.

### 4.1 Regulation of inattention by taVNS

Attention is a bottom-up process driven by external stimuli (Aniwattanapong et al., 2022). Satterfield et al. (1974) proposed a model of low arousal in the central nervous system. The essence of ADHD was primarily explored at the level of physiological arousal, which is the necessary process for maintaining normal physiological activities and alternates with the diurnal cycle (Satterfield et al., 1974). The low arousal theory suggests that children with ADHD have lower levels of arousal than neurotypical children and that their higher activity levels and stimulus-seeking behaviors may be an attempt to increase their level of arousal, thus leading them to show excessive movement to stay awake (Brennan and Arnsten, 2008). Furthermore, psychology and neuroscience suggest that vigilance and cortical arousal are important components of attentional regulation. Vigilance refers to the ability to maintain performance and stay alert, while cortical arousal represents the level of spontaneous activation in the cerebral cortex. These factors are responsible for maintaining optimal brain arousal and behavioral performance (Sherman and Usrey, 2021; Rueda et al., 2023).

The physiological basis of attention is an extensive neural network, and the neural pathways associated with this network can be further divided into three subnetworks that perform arousal, orienting, and executive control functions (Schindler et al., 2024). Using fMRI to explore the neurophysiological basis of attention, regions in the extended frontoparietal network, including the PFC, anterior cingulate cortex, anterior insular cortex, basal ganglia, and thalamus, were found to be activated in a variety of attentional functions, whereas hypoactivation of neural pathways associated with the aforementioned networks can all contribute to attention deficits (Pezze et al., 2014; Aniwattanapong et al., 2022). Increasing evidence suggests that taVNS may modulate cortical activity, excitability, and plasticity through LC-NE activation of neuromodulatory systems across a wide range of projections, including those in attentional networks (Collins et al., 2021; Chen et al., 2023a).

Furthermore, NE can control the state of cortical networks and can influence information processing (Hays et al., 2013). Salivary alpha-amylase (sAA), pupillary dilation (PD), and alpha cortical oscillations have been identified as NE markers and seem to support the existence of a close link between the taVNS and the activity of the LC-NE system (D’Agostini et al., 2023; Wienke et al., 2023). A recent large pooled analysis confirmed that taVNS stimulates NE release by analyzing 10 taVNS studies and showing that vagal activation by taVNS increased sAA release compared to sham stimulation (Giraudier et al., 2022). Wienke et al. (2023) demonstrated this link in a study of 29 subjects following separate true/false taVNS, showing that phasic taVNS significantly increased pupil dilation and improved performance during the emotional Stroop task; in magnetoencephalography, taVNS increased frontal midline theta and alpha power, reflecting a shift toward more internally oriented attention. Chen et al. (2023a) revealed that the reduction in spontaneous alpha oscillations modulated by taVNS may be associated with a broader release of inhibition and higher levels of arousal in the cortex, particularly in the sensorimotor cortex, which may further increase exogenous stimulus detection and improve executive functioning, by administering taVNS interventions to 30 subjects and no interventions to another 30 subjects, respectively. This suggests that taVNS reliably induces arousal reflected in pupillary and EEG markers beyond the effects of somatosensory stimulation, thus supporting the idea that endogenous NE-mediated arousal is dependent on the potency of exogenous stimulation with taVNS (Lerman et al., 2022).

Hence, it can be inferred that, although further clinical and basic experimental validation is required based on these findings, taVNS appears to ameliorate attentional control in ADHD patients by activating the LC-NE pathway, modulating NE release, and thereby influencing neuroregulatory systems closely associated with changes in arousal states.

### 4.2 Modulation of executive function by taVNS

Executive function (EF) deficits serve as an endophenotype of ADHD symptoms, and the hyperactive, impulsive, and inattentive behaviors exhibited by ADHD patients are often due to impaired EF (Li et al., 2023). EF encompasses three core components: inhibitory control, WM, and cognitive flexibility. Moreover, the results of a recent meta-analysis suggest that taVNS can enhance cognition, especially executive function (Ridgewell et al., 2021).

#### 4.2.1 Inhibitory control

Inhibitory control is a core executive function that is essential for adaptive behavioral regulation by inhibiting inappropriate responses (Zhu et al., 2024). PFC circuits are widely recognized as a central neural network for inhibitory control, particularly involving the inferior frontal gyrus (IFG) (Dos Santos Afonso Junior et al., 2023). In everyday life, inhibitory control enables us to control automatic impulses at the perceptual, cognitive, and behavioral levels, and its impairment is characterized by behavioral impulses (Aron and Poldrack, 2005). Therefore, improving inhibitory control is a very promising therapeutic target for clinical applications in the treatment of ADHD.

Cumulative evidence from brain imaging studies suggests that taVNS has a positive “exogenous” online neuromodulatory effect on inhibitory control, and that neural activity in the LC-NE system modulates functional connectivity within the PFC inhibitory control network (Barcomb et al., 2022). Functional MRI studies of ADHD patients have shown abnormal neural activity in the executive control and dorsal attention networks (Francx et al., 2015). And taVNS appears to repair this abnormality. Furthermore, Zhu and colleagues found that taVNS improved the accuracy of No-Go responses compared to sham stimulation; a generalized linear model analysis further revealed increased bilateral inferior frontal gyrus (IFG) activity, particularly on the right side—linked to inhibitory control—suggesting a beneficial effect of taVNS on this function (Zhu et al., 2024). Similarly, Beste et al. (2016) reported enhanced response inhibitory control following active taVNS. These preclinical studies in healthy populations have demonstrated the beneficial effects of taVNS on executive behavior and inhibitory control, further suggesting that it may have a potential therapeutic role in improving impulse control in ADHD patients.

#### 4.2.2 Working memory

Working memory is a core component of higher cognitive functioning and combines attentional control with temporary storage and information manipulation (Mukherjee et al., 2021; Sun et al., 2021). WM impairments are a typical cognitive abnormality in individuals with ADHD, and this disorder has been shown to persist into adulthood, despite the lack of effective treatment methods (Levy and Farrow, 2001).

To date, several studies have confirmed that taVNS may be a potential therapeutic intervention for impairments in the cognitive domain (Broncel et al., 2020; Wang et al., 2022; Wang Y. et al., 2023). However, relatively little has been reported on taVNS for the treatment of WM. Sun and colleagues (Sun et al., 2021) found that offline taVNS enhanced spatial WM capacity in healthy adults, as compared to online taVNS and a sham surgery group, in a study examining immediate WM enhancement. In another behavioral and physiological study, Tan et al. (2024) found that vibrotactile taVNS elevated arousal levels and maintained WM function at an optimal state. taVNS is also considered a powerful intervention for acute sleep deprivation, as it enhances performance on cognitively loaded tasks (the N-back task and the psychomotor vigilance task) and improves WM (Zhao et al., 2023). Furthermore, Pan et al. (2024) found that taVNS altered neural oscillations during WM tasks and improved WM performance in patients with epilepsy.

Although all of the above studies suggest that taVNS has effects that improve WM, these effects are thought to be related to two pathways. Firstly, taVNS activates NE neurons in the LC and cholinergic neurons in the nucleus basalis, promoting the release of central system NE, acetylcholine, and serotonin, which can enhance WM capacity, by promoting neuroplasticity. Secondly, long-term potentiation (LTP), a process involving sustained synaptic strengthening leading to a long-term increase in signaling between neurons, is a major cellular mechanism for memory formation. Thus, it has been suggested that NE can be indirectly involved in the memory formation process by facilitating the LTP effect (Lim et al., 2010). taVNS enhances memory formation by increasing NE concentration in the brain, activating β-noradrenergic receptors, and reducing hyperpolarization of hippocampal dentate gyrus neurons. The aforementioned process concurrently promotes the formation of multiple action potentials and enhances LTP effects at synapses in the dentate gyrus and other brain regions. This process, which is thought to be induced by taVNS-activated LC-NE release, has been proposed as another possible mechanism for modulating memory performance (Harley, 1991; O’Dell et al., 2015).

#### 4.2.3 Cognitive flexibility

Cognitive flexibility denotes the cognitive capacity to switch from one behavioral and mental pattern to another, primarily manifested as the ability to adjust individual behavior in response to changing environmental conditions (Zhu et al., 2023). Deficits in cognitive flexibility may lead to the persistent exhibition of maladaptive and disruptive behaviors among children with ADHD. Cognitive flexibility can be categorized into several different forms (e.g., set-shifting, reversal learning, and suppression control), all of which are heavily dependent on the PFC and can be modulated by monoaminergic and cholinergic afferents (Altidor et al., 2021). Thus, pharmacological manipulations targeting these neurochemical systems could be a therapeutic strategy to enhance cognitive flexibility.

Norepinephrine neurons in the LC innervate the PFC and the hippocampus, while NE signaling in these structures influences many forms of cognition, including cognitive flexibility. Even though early studies suggested that LC-NE system activity increases the signal-to-noise ratio and improves the brain’s ability to process sensory inputs, it reduces the brain’s ability to recruit large-scale networks and may be responsible for the VNS impairing cognitive flexibility and creative thinking (Ghacibeh et al., 2006). However, emerging evidence seems to reverse this claim. By administering tVNS to 32 participants, Borges et al. (2020) showed that tVNS increases cognitive flexibility in a set-shift paradigm and that tVNS may have a stronger effect on cognitive flexibility than inhibition. Another study demonstrated that acute VNS facilitated reversal learning and showed that the timing and frequency of VNS were critical for these enhancement effects (Altidor et al., 2021). In addition, Driskill et al. (2022) found that VNS enhanced cognitive flexibility, further proposing that the optimal functioning of the PFC-subcortical inhibitory circuitry was reflected in VN-mediated HRV, i.e., higher resting-state HRV promoted cognitive flexibility in human subjects. Similarly, studies in animal models have shown that rats given the NE reuptake inhibitor AXT enhanced reversal learning in the same task (Altidor et al., 2021). Thus, VNS can enhance cognitive flexibility, suggesting its potential use in alleviating inattentive symptoms in children and adults with ADHD, while the evidence against taVNS for improving cognitive flexibility still needs to be further studied.

## 5 Conclusion

Despite the gradual increase in research on taVNS over the past two decades, the field is still in its infancy. Currently, NE-related pathways have been identified as key targets for taVNS to exert its therapeutic effects (Ludwig et al., 2021; Chen et al., 2023b), and we have identified the potential use of taVNS in patients with ADHD by citing evidence related to the activation of the NST-LC-NE-PFC system by taVNS. However, there are still some issues that we need to consider: (1) taVNS has been proposed in existing studies to treat neurological and psychiatric disorders through multiple pathways, such as cholinergic anti-inflammatory pathway (Liu et al., 2024), excitatory or inhibitory pathways of neurotransmitters (γ-aminobutyric acid, 5-hydroxytryptophan, and DA) (Kim et al., 2022; Li et al., 2022), and epigenetics to regulate neuroplasticity and promote functional recovery. The manuscript only explores the hypothesis of the mechanism of action of taVNS in treating ADHD through the NE system. Additionally, taVNS may also exert a therapeutic effect on ADHD through other pathways, and other biomarkers need to be proposed in the future to verify the mechanism of action. (2) While most ADHD patients are children or adolescents, the evidence population discussed in the article is mostly adults, and it remains unknown whether taVNS will produce the same therapeutic effect in children or adolescents compared to adults. (3) Considering that taVNS has not yet been effectively disseminated within the ADHD patient population, further research is necessary to determine the optimal taVNS stimulation parameters for ADHD patients and to investigate whether they exhibit dose-dependency to this treatment. Furthermore, despite the widespread reporting of taVNS’s safety (Kim et al., 2022; Tan et al., 2023), clinical observational studies are still needed to strengthen the evidence regarding potential off-target effects and adverse reactions associated with its use in treating ADHD patients.

In conclusion, taVNS is more likely to be accepted by ADHD patients and their parents as a non-invasive neuromodulation technique that is easy to administer, easily accessible, and has fewer side effects. The evidence presented here intends to demonstrate that taVNS potentially operates via endogenous subcortical neuromodulatory signals. These signals transmit peripheral information to the central nervous system, activate the NE system, and enhance neuronal activity within the brain. Consequently, this process modifies the function of relevant brain regions, primarily the prefrontal cortex, and plays a crucial role in regulating attention, arousal, cognition, and behavior—key aspects targeted for improvement in ADHD treatment.
